# Supplementary material for: Discovery and structural analysis of glycoside hydrolase family 176 α-1,2 glucosidase from Arthrobacter humicola A8F5
Source: J Biol Chem. 2026 Sep 8;302(10):113530. doi: 10.1016/j.jbc.2026.113530 (PMC13671375; doi:10.1016/j.jbc.2026.113530)
Supplement: Supplementary file 1 [file mmc1.pdf]

Discovery and structural analysis of glycoside hydrolase family 176 α-1,2 glucosidase from *Arthrobacter humicola* A8F5

Ryota Yasukochi (安河内亮太)[1,#], Takashi Suzuki (鈴木貴視)[2,#], Terumasa Toraya (虎谷輝正)[2], Keiko Hino (日野恵子)[2], Tetsuya Mori (森哲也)[2], Toma Kashima (鹿島騰真)[1,3], Akimasa Miyanaga (宮永顕正)[1,3], Hikaru Watanabe (渡邊光)[2*], Shinya Fushinobu (伏信進矢)[1,3*]

[1]Department of Biotechnology, The University of Tokyo, Tokyo, Japan; [2]Research&Development Division, Nagase Viita CO., LTD., Okayama, Japan; and [3]Collaborative Research Institute for Innovative Microbiology, The University of Tokyo, Tokyo, Japan

[#]These authors contribute equally to this study.

[*]Correspondence to: Shinya Fushinobu, asfushi@mail.ecc.u-tokyo.ac.jp; Hikaru Watanabe, hikaru.watanabe@nagase.co.jp

List of materials included

- **Tables S1–S3**
- **Figures S1–S4**

Table S1. Effects of metal salts on the A8F5 glucosidase activity

**Metal salts or chelator**	**Relative activity (%)**	**Metal salts**	**Relative activity (%)**
None	100.0	$MnCl_2$	100.1
EDTA	95.5	$CoCl_2$	96.1
RbCl	99.1	$NiCl_2$	86.1
KCl	97.9	$PbCl_2$	101.0
NaCl	100.9	$SnCl_2$	97.5
NH_4Cl	100.6	$SrCl_2$	100.6
LiCl	101.0	$CaCl_2$	101.5
$MgCl_2$	100.6	$FeCl_2$	96.2
$BaCl_2$	102.1	$ZnCl_2$	88.2
$HgCl_2$	1.2	$AlCl_3$	97.2
$CuCl_2$	3.4	$FeCl_3$	95.1

Enzyme activity was measured in the presence of metal salts or a reagent (1 mM) under the optimal condition (pH 6.5, 40°C). EDTA: ethylenediaminetetraacetic acid.

Table S2. Crystallographic data collection and refinement statistics of A8F5 glucosidase

	WT + glucose	Δloop1 ligand-free	Δloop1 D427N + kojibiose	Δloop1 D427N + kojitriose	Δloop1 D427N + selaginose
**Data collection**[a]					
Beamline	PF AR-NE3A	PF BL-1A	SPring8 BL45XU	SPring8 BL45XU	PF BL-5A
Wavelength (Å)	1.0000	1.0070	1.0000	1.0000	1.0000
Space group	$P2_12_12_1$	$P2_1$	$P2_1$	$P2_1$	$P2_1$
Unit cell *a*, *b*, *c* (Å)	104.5, 107.1, 110.4	55.9, 109.9, 105.2	55.8, 109.5, 105.4	56.4, 108.0, 105.5	55.8, 108.1, 105.4
α, β, γ (°)	90, 90, 90	90, 93.66, 90	90, 93.27, 90	90, 93.34, 90	90, 93.28, 90
Resolution (Å)	49.07-1.90 (1.93-1.90)	48.70-1.88 (1.91-1.88)	49.64-1.79 (1.82-1.79)	48.49-1.83 (1.86-1.83)	49.52-1.80 (1.83-1.80)
Total reflections	648415 (30699)	363408 (18350)	1351538 (202499)	1570649 (75995)	375181 (18869)
Unique reflections	98048 (4791)	103014 (5090)	119031 (18995)	111405 (5305)	114361 (5627)
Multiplicity	6.6 (6.4)	3.5 (3.6)	11.4 (10.7)	14.1 (14.3)	3.3 (3.4)
Completeness (%)	100.0 (100.0)	100.0 (100.0)	99.8 (99.1)	99.8 (96.9)	99.1 (99.7)
Mean $I/\sigma(I)$	7.9 (1.7)	5.0 (1.5)	14.21 (2.36)	7.8 (1.0)	14.3 (2.1)
R_{merge}	0.102 (1.136)	0.175 (0.928)	0.226 (1.495)	0.267 (3.160)	0.045 (0.515)
R_{meas}	0.111 (1.237)	0.207 (1.092)	0.237 (1.571)	0.277 (3.277)	0.054 (0.613)
$CC_{1/2}$	0.998 (0.621)	0.977 (0.536)	0.997 (0.796)	0.995 (0.567)	0.999 (0.848)
**Refinement**					
Resolution range (Å)	49.07-1.90	47.43-1.88	48.60-1.79	48.49-1.83	48.16-1.80
R_{work}/R_{free}	0.215/0.263	0.207/0.257	0.168/0.210	0.210/0.257	0.199/0.240
RMSD bond lengths (Å)	0.010	0.015	0.015	0.015	0.015
RMSD bond angles (°)	1.940	2.454	2.221	2.337	2.305
Mol/ASU[b]	2	2	2	2	2
Ramachandran plot (%)					
Favored	96.18	96.62	96.87	95.36	95.91
Allowed	3.57	2.97	2.72	3.89	3.98
Outlier	0.25	0.41	0.41	0.75	0.41
**PDB ID**	24IS	24IR	24AI	24AZ	24IL

[a]Values in parentheses represent the highest resolution shell. [b]Number of molecules per asymmetric unit.

Table S3. Designed loop deletants

Name	Amino acid sequence (E31–G59)
WT	ESNPPGARQSGQLAGQSGDPAGGSTDPAG
Candidate 1	ESNPPGA---------------------G
Candidate 2	ESNPPGAR--------------------G
Candidate 3	ES---------------------STDPAG
Candidate 4	ES--------------------GSTDPAG
Candidate 5	ES--------GSSGSS------------G
Candidate 6	ES--------GSSSSG------------G
Candidate 7	ES--------GSSSG-------------G
Candidate 8	ES--------SSSSS-------------G
Candidate 9	ES--------GGGGG-------------G
Candidate 10	ESNPPG----------------------G
Candidate 11	ESNPP-----------------------G
Candidate 12	ESNP------------------------G
Candidate 13	ES--------SGS---------------G
Candidate 14 (Δloop1)	ES--------GGS---------------G
Candidate 15	ES--------GSGS--------------G

Candidate 14, featuring a GGS linker, was selected as Δloop1 construct after in *silico* screening.

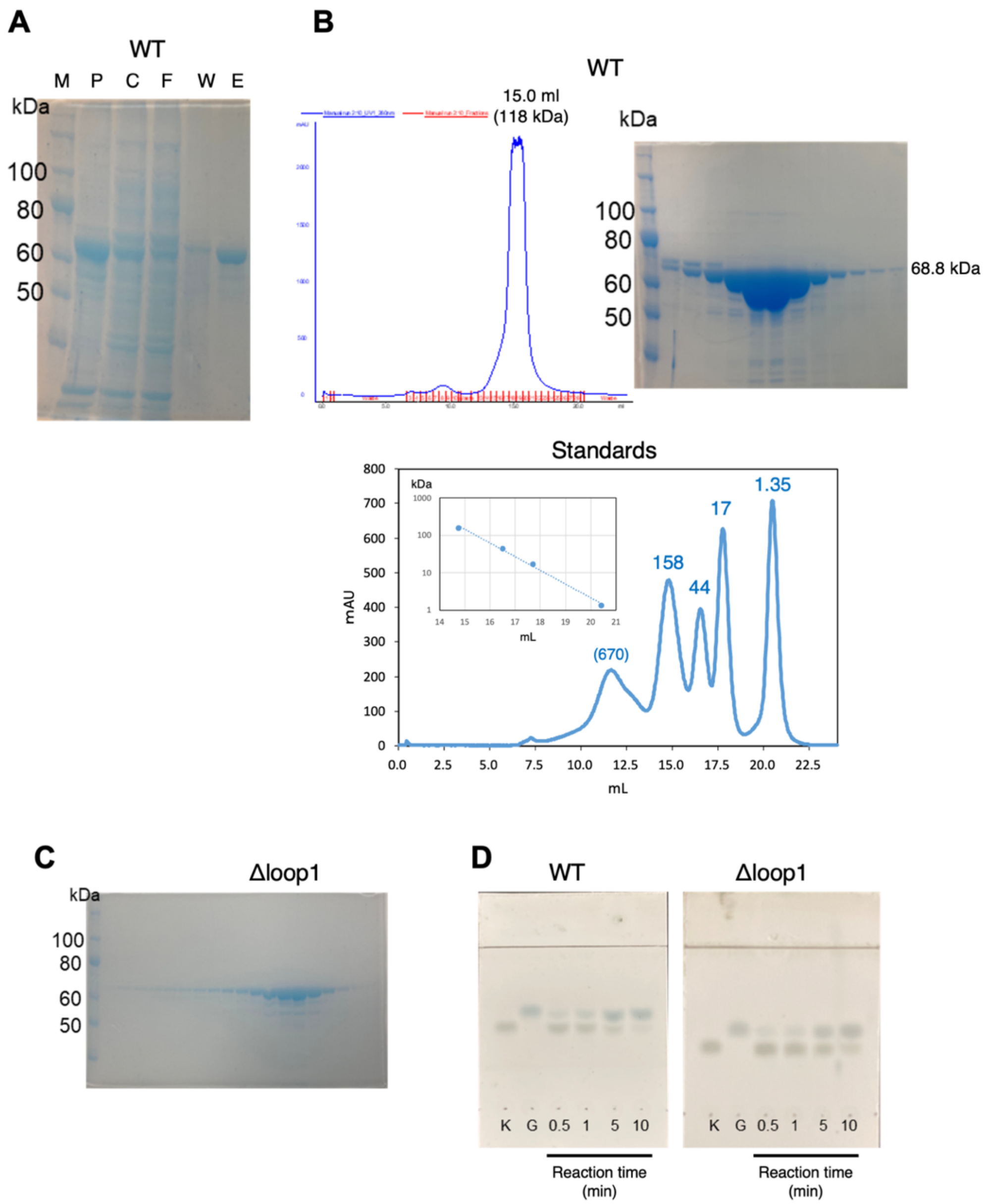

Figure S1. Purification and activity of recombinant proteins of wild-type (WT) A8F5 glucosidase and the Δloop1 construct. *A*, SDS-PAGE of each fraction before and after nickel affinity chromatography during WT protein purification. Symbols: M, molecular size marker; P, pellet; C, crude supernatant; F, flow-through; W, wash; E, eluate. *B*, Gel filtration chromatography for WT protein purification using a Superose 6 Increase 10/300 GL column. The chromatogram of WT A8F5 glucosidase (top left), SDS-PAGE of the collected eluate fractions (top right), and the chromatogram of molecular weight standards (bottom) are shown. *C*, SDS-PAGE of the collected eluate fractions from gel filtration chromatography of Δloop1. *D*, TLC analysis on the activity of WT A8F5 glucosidase (left) and Δloop1 (right). Symbols: K, 1 mM kojibiose; G, 1 mM glucose; 0.5–10, reaction time (min). SDS-PAGE gels were stained by Coomassie Brilliant Blue.

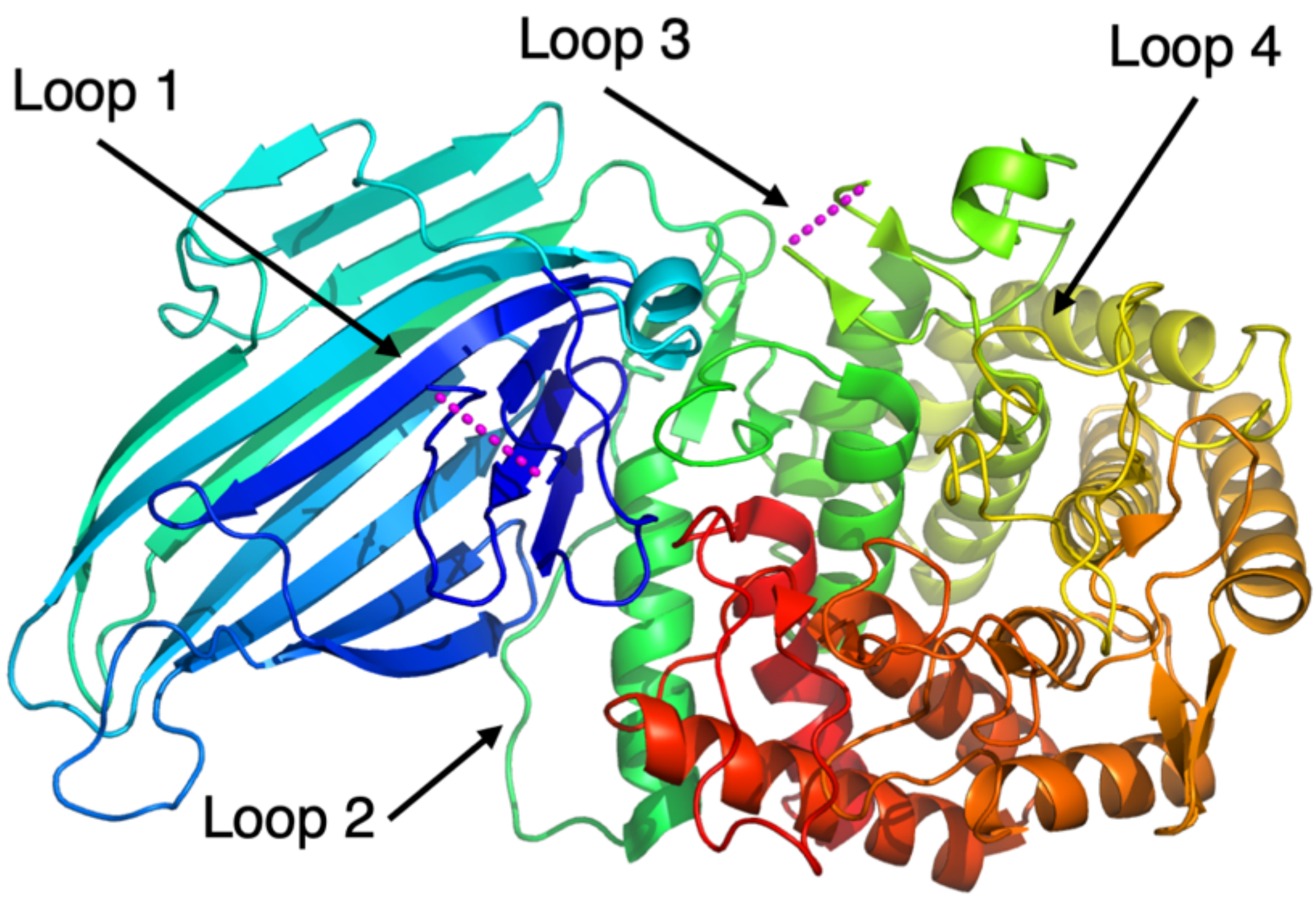

Figure S2. Monomer structure of wild-type A8F5 glucosidase (chain B). The overall structure of the chain A monomer is shown in Fig. 3*C*.

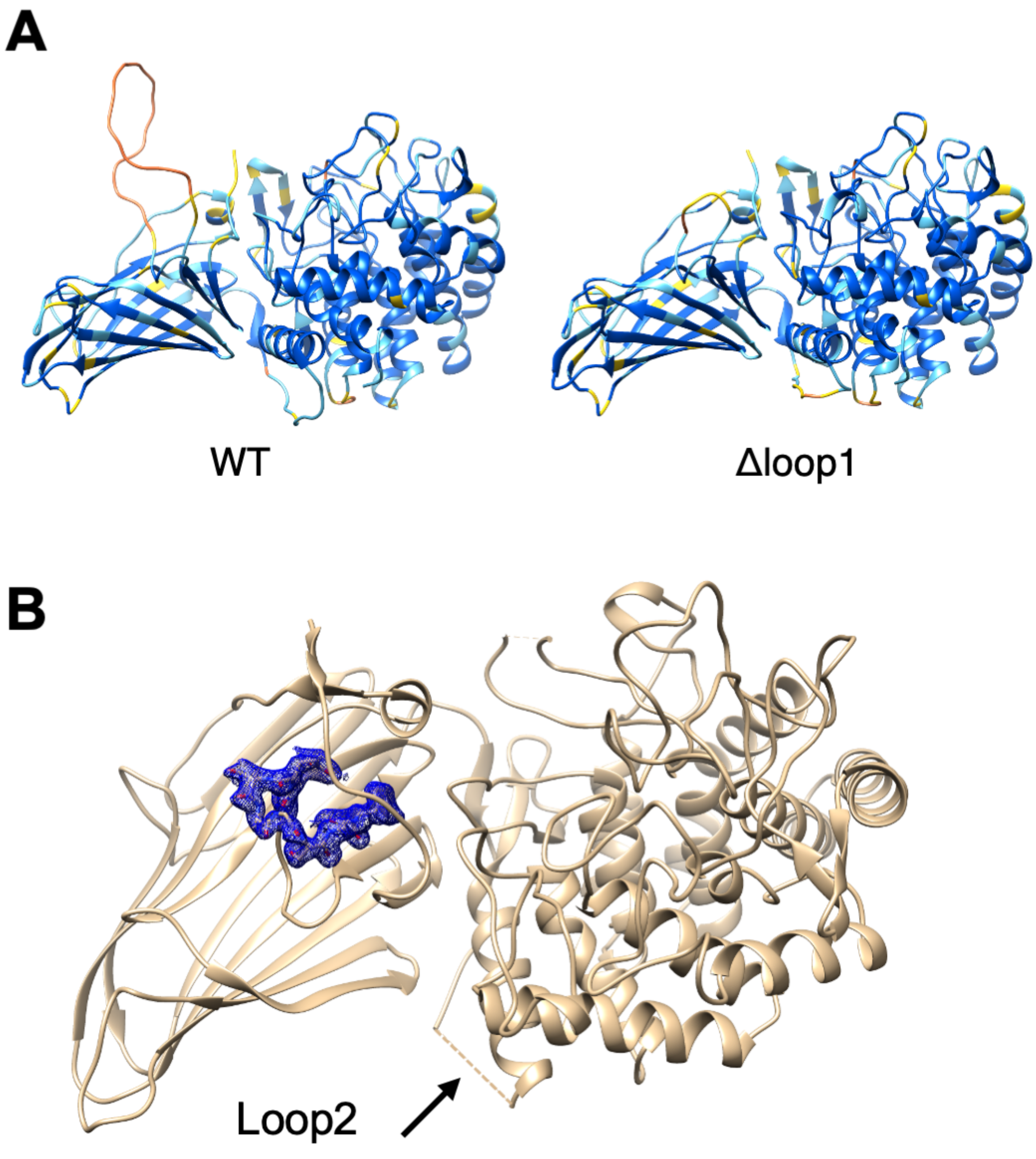

Figure S3. Structure-based design and validation of the Δloop1 construct. *A*, AlphaFold3-predicted structures of the WT (left) and Δloop1 (right) colored by pLDDT from low (orange) to high (blue). *B*, The ligand-free crystal structure of the Δloop1 construct. Polder map for the engineered linker region (GESGGSGF) is shown as a blue mesh contoured at 3σ. In the Loop 2 region, the electron density was too weak to build a model.

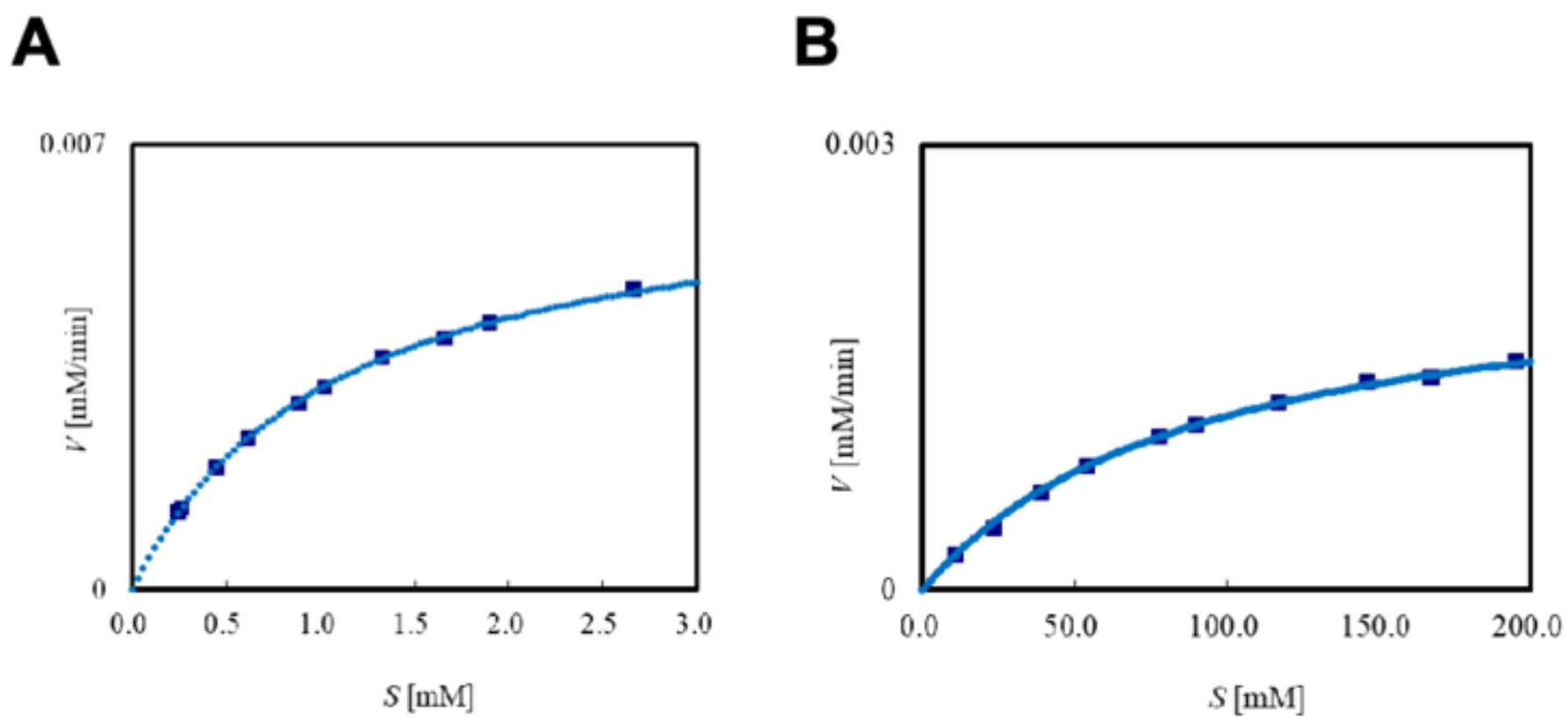

Figure S4. *S*-*v* plots used for kinetic parameter determination. Plots for kojibiose (*A*) and nigerose (*B*) are shown.
